# The biogeography of soil and airborne fungi in the Southwestern USA in relation to climate and vegetation

**DOI:** 10.1093/ismeco/ycaf249

**Published:** 2026-01-09

**Authors:** Linh Anh Cat, Morgan E Gorris, James T Randerson, Stephanie N Kivlin, Kathleen K Treseder

**Affiliations:** National Park Service, Kalaupapa National Historic Park, Honolulu, HI 96850, United States; Los Alamos National Laboratory, Information Systems and Modeling Group (A-1), Los Alamos, NM 87545, United States; Department of Earth System Science, University of California, Irvine 92697, United States; Department of Ecology and Evolutionary Biology, University of Tennessee Knoxville, Knoxville, TN 37996, United States; Department of Ecology and Evolutionary Biology, University of California, Irvine, CA 92697, United States

**Keywords:** dispersal, distance-based redundancy analysis, distance–decay relationships, fungal community composition, precipitation, spatial autocorrelation

## Abstract

To assess how fungal dispersal might respond to climate change, we examined how climate and geography influence the regional distribution of fungi in soil and air. Specifically, we hypothesized that neighboring fungal communities should be more similar than distant communities (i.e. spatially autocorrelated) and that fungal dispersal should be more limited in soil than in air. We collected soil and air samples from 60 sites across five states in the Southwestern USA. Then, we sequenced the ITS2 region to identify fungal taxa in each sample. Next, we used distance-based redundancy analysis to partition variation in fungal community composition between climate variables and spatial structure. Fungi were indeed spatially autocorrelated. Moreover, precipitation, maximum vapor pressure deficit, and soil moisture were significantly related to fungal community composition in soils. In comparison, only precipitation was significantly related to community composition in the air. After accounting for climate, the strength of spatial autocorrelation did not differ significantly in soilborne versus airborne fungi. Dispersal limitation was evident in soilborne fungi at short distances (<100 km) and was not observed at any distance in airborne fungi. Altogether, climate may influence which fungal taxa are present in soil and air, and fungi could feasibly wind disperse over regional scales.

## Introduction

Soil fungi can respond to climate change with acclimation [1], shifts in community composition [2], evolution [3], or, potentially, dispersal [4]. Climate change may influence fungi in warm, arid regions such as the Southwestern USA [5–7]. Could fungi disperse via wind to follow their climate niches? To answer this question, we need to understand how climate relates to fungal biogeography in the soil and air in this region. The answer can help us determine how easily fungi could readily disperse, and to what extent environmental conditions are important [8–10].

Climate change is altering the frequency and severity of drought and heat waves in the Southwestern USA [11, 12]. Specifically, climate models predict that this region should experience longer, more severe droughts interspersed with larger, less frequent rainstorms [12–14]. Overall, mean annual precipitation may decline up to 25 mm y^−1^ by mid-century [13, 15]. In addition, mean annual temperatures are expected to increase 1.1°C–2.2°C by mid-century [14, 16]. These projections are consistent with empirical trends documented in this region over the past several decades [14, 17–19].

These trends can influence fungal ecology, since taxa vary in their sensitivity to water availability and temperature [20–26], and climate thresholds can define the ecological niche of fungi [27]. For example, *Coccidioides*, a fungus that causes valley fever in the Southwestern USA, is most commonly detected in dry areas with a mean annual temperature >11°C [28]. Accordingly, Gorris *et al.* [4] predicted that climate change would expand the potential geographic range of *Coccidioides* to the Northern USA by the end of this century. Winds readily carry the spores of this fungus [29], so it could potentially disperse to these new areas [4]. Other fungi might be likewise poised to disperse.

Indeed, soil fungal communities often covary with water availability and temperature across the landscape [6, 9, 30–33]. Specifically, these patterns have been observed at the regional scale (>1000 km distance) in Spain [34], Switzerland [34, 35], western China [36], and Tibet [37]. In the Southwestern USA, the climate varies from hot and dry to cool and mesic as a function of elevation and the influence of rain shadows [38]. These climate gradients could contribute to fungal biogeography there.

As climate shifts, how easily might fungal taxa follow their climate niche? Dust storms are increasing in frequency in the Southwestern USA, owing to anthropogenic soil disturbances such as off-road vehicle use, construction, road maintenance, military activities, grazing, and agriculture [39]. Dust storms can entrain spores in the air and disperse them over relatively long distances [40, 41]. In addition, many fungal species actively launch spores into the air [40, 42]. About half of fungal species produce fairly small spores—<10 m diameter at their longest axis [43]. For example, *Aspergillus, Penicillium,* and *Cladosporium* species have an aerodynamic diameter around 5 m and are commonly detected in air samples [44]. Species in this size range are relatively likely to be wind transported [5, 45] since dust particles smaller than 10 m in diameter can remain airborne long enough to travel significant distances [46]. In fact, a global spore sampling project collected about 28 000 fungal species from the air in 47 sites [47]. Moreover, some fungal spores can be particularly resistant to UV radiation and desiccation, such as those with melanin [48–52]. At an extreme, at least some fungi can remain viable in the atmosphere long enough to cross continents or oceans [53, reviewed in 54, 55, 56]. However, it remains unknown how far they typically disperse and under which environmental conditions.

We can examine distance–decay relationships across the region to determine the extent to which climate and dispersal influence fungal communities in the soil and air [8, 9, 57]. Distance–decay relationships describe how communities become dissimilar as the distance between them increases [57–59]. This pattern can be caused by dispersal limitation, environmental heterogeneity, and ecological drift (e.g. [8, 60, 61]). With all else being equal, environments that facilitate dispersal over long distances should display a weaker distance-decay relationship than those in which dispersal is restricted. Accordingly, Clark *et al.* [57] proposed that distance–decay relationships in airborne fungi should be weaker than those in soilborne fungi. In other words, spatial autocorrelation of community composition should be less significant in the air than in the soil.

We tested three hypotheses by sampling soil and air communities across five states within the Southwestern USA. First, we hypothesized that fungal richness and community composition in soils of the Southwestern USA should be related to climate or vegetation (H1). Second, across the region, fungal communities that are closer to one another should be more similar than those that are more distant (H2). We expect this because fungi should disperse more readily among neighboring communities, and environmental conditions tend to be spatially autocorrelated. Third, the spatial autocorrelation of airborne fungal communities should be weaker than that of soilborne fungal communities, owing to wind dispersal of fungi (H3).

## Materials and methods

### Field sites

**Figure 1:**
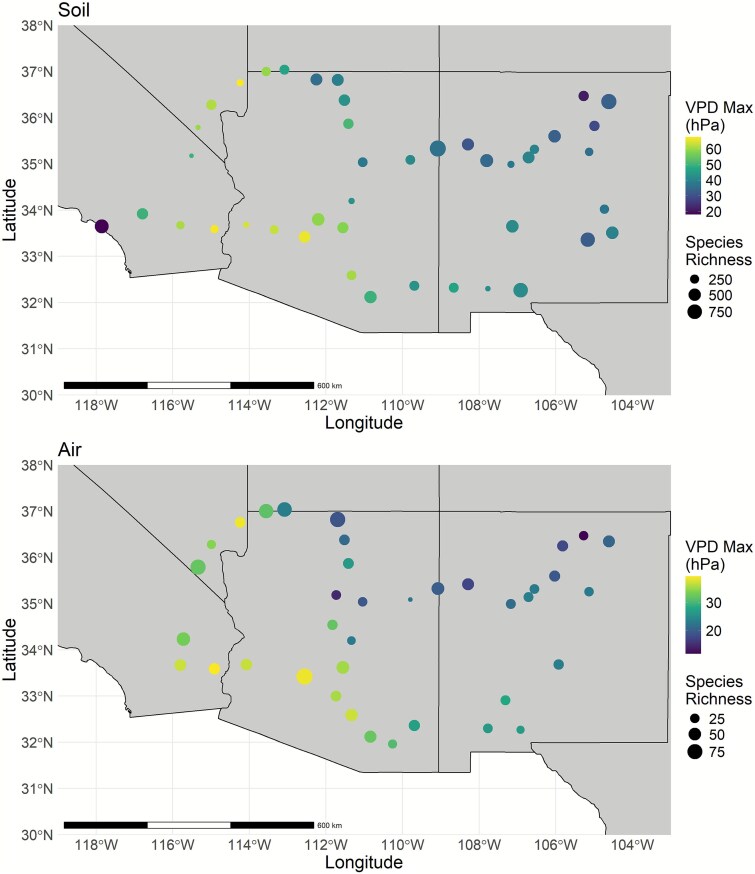
Spatial structure of fungal richness in soil and air samples in the Southwestern USA. Symbols are sites. Symbol size represents the number of taxa detected. Symbol color represents the maximum VPD.

We conducted our study in the US southwestern region, covering ~86 000 km^2^. Over 5 days in mid-July 2015, we collected soil and air samples at 60 sites across Arizona, California, Nevada, New Mexico, and Utah. We selected sites that followed approximate north–south and east–west transects and were at least 1 km from major highways (Table S1, Fig. 1). We selected sites at driving intervals of ~100 km. We used a global positioning system to record latitude, longitude, and elevation at each site.

### Climate

We quantified mean annual temperature and mean annual precipitation from August 2014 through July 2015, and maximum vapor pressure deficit (VPD) from July 2015, for each site using data from the PRISM Climate Group at Oregon State University [62]. Each of these ranges overlapped with the sampling period. We selected maximum VPD because it is a particularly physiologically stressful condition.

### Vegetation

We extracted Enhanced Vegetation Index (EVI) data for each of our soil and air sample locations. The EVI provides a measure of vegetation “greenness” after correcting for atmospheric scattering from aerosols and haze. Theoretically, the values can range from −1 to 1, but measured values typically range from 0 to 0.9; values 0.0–0.2 are likely barren or desert, values 0.2–0.4 are sparse vegetation or grasslands, values 0.4–0.6 are moderate vegetation, and values above 0.6 are dense vegetation. We downloaded the July 2015 EVI data for our study region available at 1 km resolution from the MODIS/Terra Vegetation Indices Monthly L3 Global 1 km SIN Grid V061 dataset (MOD13A3 v061; ref. [63]). We also extracted the primary land cover type for each of our sample locations from the 2015 National Land Cover Data, which is available each year at 30-m resolution and contains 16 major land cover classes [64, 65].

### Sample collection

Within each site, we collected a 2.5 cm-diameter × 10 cm deep soil core from each of two random locations within ~20 m of one another (processed independently prior to data analysis). As we were examining regional-scale phenomena instead of fine-scale edaphic variation, we invested sampling effort at the regional scale rather than the local scale. Random locations were selected regardless of vegetation coverage, so some were near vegetation and others were in bare ground. We deployed a Biostage air sampler (SKC, Inc., Eighty Four, PA) to capture air samples from the midpoint between soil sample locations. With this technique, fungal spores as small as ~0.6 μm were captured on plain agar (with no nutrients) in a Petri dish. The sampler ran for 5 min at a sampling rate of 20 l/min, for a total of 100 l of air sampled. We kept air and soil samples on dry ice during the duration of the sampling trip, and stored the samples at −80°C upon return to the lab. In total, we collected 120 soil samples and 60 air samples.

### Soil properties

We measured soil pH on a 1:1 ratio (w/v) of soil to deionized water. We determined soil moisture gravimetrically by drying subsamples for 48 h at 65°C. We measured soil salinity as the conductivity of a 1:5 w/v soil to deionized water mix.

### DNA extraction

We processed air and soil samples using the MoBio PowerSoil Extraction kit (MoBio, Carlsbad, CA). For soil samples, we extracted DNA from 250 mg of soil according to the manufacturer’s recommendations. For air samples, we removed a sub-sample of the agar from a 2 cm by 2 cm surface of the Petri dish, and then followed the manufacturer’s directions for all other steps. Both soil and air samples tended to be too dilute for effective PCR amplification, so they required concentrating using the Zymo DNA Concentrator kit (Zymo Research Corp., Irvine, CA). We standardized DNA concentrations to 10 ng/μL before PCR.

### PCR amplification and sequencing

We amplified a 340 bp region of the fungal ITS2 region in the 5.8S encoding gene. This shorter amplicon reduces species bias and chimera formation without compromising inferences of species diversity [66]. We used a staggered primer design to improve overall sequence quality in the Illumina MiSeq flowcell as described in Looby *et al.* [67, 68]. The forward primer (ITS9f; AATGATACGGCGACCACCGAGATCTACAC TC TTTCCCTACA CGACGCTCTTCCGATCT NNNNNGAA CGCAGCRAAIIGYGA) was standard across all samples, and the reverse primer (CAAGCAGAAGACGGCATACGAGAT) also had a 12 bp barcode to identify unique samples, pad (AGTCAGTCAG), linker sequences (CC), and the ITS4 primer (TCCTCCGCTTATTGATATGC). These are universal fungal primers, although they have a known bias against fungi in the Glomeromycota [69–71].

Reactions contained 0.75 ng of each forward and reverse primer (10 μmol L^−1^), 21.5 μL of Platinum PCR SuperMix (Invitrogen, Carlsbad, CA), 1 μL of BSA (10 mg mL^−1^), and ~10 ng of template DNA for a total volume of 25 μL. We amplified each sample in triplicate, and the reaction ran for 35 cycles of 94°C for 45 s, 50°C for 1 min, and 72°C for 90 s with a hot start at 94°C for 7 min and a final extension step at 72°C for 10 min. Tedersoo *et al.* [72] recommends using fewer than 30 PCR cycles unless amplification is weak, as was the case for the air samples. We added five extra PCR cycles to all samples to maintain consistency. We pooled and purified triplicates to remove primer-dimers and non-target DNA using Agencourt AmPure XP beads (Beckman-Coulter, Brea, CA) mixed with PCR product at a 1:1 ratio. To create a multiplexed library, we quantified DNA concentrations with a spectrophotometer and the Qubit dsDNA High Sensitivity Assay Kit (Life Technologies, Grand Island, NY) and then pooled samples in equi-molar concentrations. Samples were sequenced at the University of California, Riverside Institute for Integrative Genome Biology on the Illumina Mi-Seq platform on a single flowcell lane at 2 x 300 bp paired end reads.

We obtained ~8.6 million total sequences from one lane of the Mi-Seq run. Using the open-source Quantitative Insights into Microbial Ecology (QIIME) pipeline, raw sequence data were demultiplexed [73]. We filtered reads using the following parameters: (i) a minimum Phred quality threshold of Q30, (ii) elimination of sequences with three consecutive low-quality base reads, and (iii) a minimum ratio of 0.75 for high-quality base calls to input read length. We removed chimeras by comparing against reference sequences for fungi from the UNITE database, the most comprehensive database for fungal taxa (v.7, updated 01/31/2016, https://unite.ut.ee/repository.php) [74]. Following open reference picking of OTUs by 97% similarity, OTUs were assigned taxonomy using BLAST (version 2.2.22) and the UNITE database. The 97% cutoff is equivalent to species or genus [75]. To reduce noise from sequencing errors or chimeras, we removed global singletons. After taxonomical assignment, non-fungal OTUs were filtered out. Following quality control, our dataset contained ~2.1 million fungal sequences. We used QIIME to rarefy samples to standardize the number of sequences for each site through random sub-sampling. Rarefaction levels differed between soil and air samples, because soil samples had higher sequence reads than air samples. To use data from most of our air samples, we rarefied at a lower level. We used samples from 51 out of 60 sites for soil, and 40 out of 60 sites for air. Some samples were not used because PCR amplification failed or produced substantially fewer sequences than the remaining samples. Rarefaction levels were as follows: 5000 sequences for analyses of soil communities, and 400 sequences for analyses of air communities. After rarefaction, sequence reads from soil samples that were collected in duplicate were averaged between samples from the same site. Specifically, we averaged the relative abundance of each taxon within each site. We averaged the samples to avoid overcomplicating the statistical models, which were relatively elaborate. We acknowledge that we lost some statistical power as a result. Because our fungal primers are biased against Glomeromycota [69–71, 76], we omitted sequences from this phylum from our analyses.

### Statistics

To assess the contributions of climate and—for soil samples only—soil conditions on richness, community composition, and individual fungal taxa, we used a distance-based redundancy analysis (dbRDA) accounting for spatial autocorrelation with distance-based Moran’s eigenvector maps (dbMEM) [77–79]. This approach allowed us to partition variance between geographic and environmental factors. In other words, we could isolate the effects of climate, vegetation, or soil conditions while taking spatial factors into account.

In each test, we first used the *sf* package in R [80, 81] to transform geographic coordinates to a projected coordinate system (NAD83/Conus Albers) to ensure accurate spatial representation. These coordinates were then extracted to compute dbMEM variables, which represent spatial autocorrelation structures. Second, we used the “spdep” package to identify significant dbMEM variables [82–84]. Third, we conducted a dbRDA using the “capscale” function with the “vegan” package to examine environmental and spatial variables [85]. Separate dbRDA models were fitted for (i) environmental variables only, (ii) significant dbMEM variables, and (iii) a combined model with both environmental and dbMEM variables. We applied a permutation-based analysis of variance with 99 999 permutations to test the significance of variables in each model. We followed this basic framework to test Hypotheses 1 to 3. The specific variables we tested depended on the hypothesis, and we detail these below. Results were considered significant when *P* < .05 and marginally significant when *P* < .10.

Hypothesis 1. We examined taxon richness as our metric of diversity. We used the “specnumber” function within the “vegan” package in R to calculate the total number of unique taxa within each rarified sample [85]. This number was the dependent variable in a multivariate linear model including dbMEMs to account for spatial autocorrelation. For soil samples, the environmental predictors tested were soil moisture, soil pH, soil conductivity, soil salinity, mean annual precipitation, mean annual temperature, maximum VPD, EVI, and land cover category. For air samples, we tested mean annual precipitation, mean annual temperature, maximum VPD, EVI, and land cover category only. Significant effects of soil moisture, mean annual precipitation, mean annual temperature, maximum VPD, EVI, or land cover category would support Hypothesis 1 for richness.

Next, we evaluated environmental contributions to variation among samples in fungal community composition. We used the “vegan” package to calculate Bray Curtis dissimilarity matrices from the presence or absence of each taxon in each site [85] and performed an dbRDA. Independent variables were the same as for richness, above. Significant effects of climate or vegetation factors would support Hypothesis 1.

Once we had identified the climate factors that most influenced shifts in community composition across the region, we then used the “indicspecies” package to determine which taxa contributed most strongly to those shifts [86]. For both soil and air samples, precipitation was the environmental variable most strongly related to community composition (Table 1). We therefore identified taxa responding to precipitation by binning sites into two groups based on precipitation levels. We then contrasted the relative abundance of each taxon between the two bins. For air samples, land cover category was also linked to community composition. Accordingly, we used “indicspecies” to identify which taxa contributed to the variation among land cover categories.

Hypothesis 2. First, we created maps of fungal community composition across our sites to assess spatial patterns for both soil and air samples. We used the “vegan” package to perform non-metric multidimensional scaling (NMDS) with Bray Curtis dissimilarity as the response variable. To account for the effects of climate and vegetation on community turnover, we removed the influence of mean annual precipitation, mean annual temperature, maximum VPD, EVI, and land cover category from the NMDS scores using linear regression. We then mapped the residuals of the first NMDS axis for both soil and air samples. These residuals represent the variation in community composition not explained by the selected climate variables.

**Table 1:** Statistical results for taxon richness and community composition.^†^

	Richness					Community composition			
	Soil		Air			Soil		Air	
	F ratio	P	F ratio	P		F ratio	P	F ratio	P
Mean annual precipitation	2.400	0.143	2.101	0.165		2.403	**< 0.001**	2.084	**0.008**
Maximum VPD	8.911	**0.011**	3.177	0.091		1.457	**0.029**	1.475	0.072
Mean annual temperature	4.397	0.054	0.057	0.813		1.796	**0.006**	1.559	0.051
Enhanced vegetation index	0.227	0.637	0.019	0.893		1.128	0.249	1.408	0.092
Land cover category	1.045	0.426	1.528	0.228		0.970	0.600	1.306	**0.029**
Soil moisture	0.166	0.690	N/A	N/A		1.469	**0.037**	N/A	N/A
Soil pH	0.305	0.589	N/A	N/A		1.331	0.072	N/A	N/A
Soil salinity	0.150	0.696	N/A	N/A		1.284	0.094	N/A	N/A

^†^Significant effects in bold (*P* < .05), N/A = not applicable.

Next, to assess spatial autocorrelation in these residuals, we performed a Mantel test. Specifically, we checked for correlations between the NMDS1 residuals and geographic distance. We ran separate analyses for soil versus air samples. A significant positive Mantel r for each would support Hypothesis 2.

Hypothesis 3. For this hypothesis, we started by comparing the strength of any spatial autocorrelation for soilborne versus airborne fungi across all sites. The test was performed using Pearson’s correlation method with 99 999 permutations to assess significant differences between the Mantel r statistics.

Next, we explored finer geographic resolutions in potential autocorrelation. Distance intervals between pairs of sites were defined in 100 km bins. For each interval, a Mantel test was performed using bootstrapping (1000 iterations) to estimate Mantel’s r statistic with 95% confidence intervals. In this way, we could assess the strength and direction of Mantel correlations for sites located within 100 km of one another, those within 100 to 200 km, within 200 to 300 km, and so on. Hypothesis 3 would be supported if the 95% confidence intervals of Mantel r values did not overlap between air samples and soil samples.

## Results

We observed a total of 7583 fungal taxa among the 220 000 soil sequences and 668 taxa in the 16 000 air sequences. Pleosporales dominated, representing 32% of soil sequences and 46% of air sequences (Fig. S1). In soil samples, Agaricales and Sordariales were also common. In air samples, Tremellales and Hypocreales were particularly abundant. Altogether, the taxa represented a broad selection of the fungal tree of life.

### Hypothesis 1: Richness and community composition

**Figure 2:**
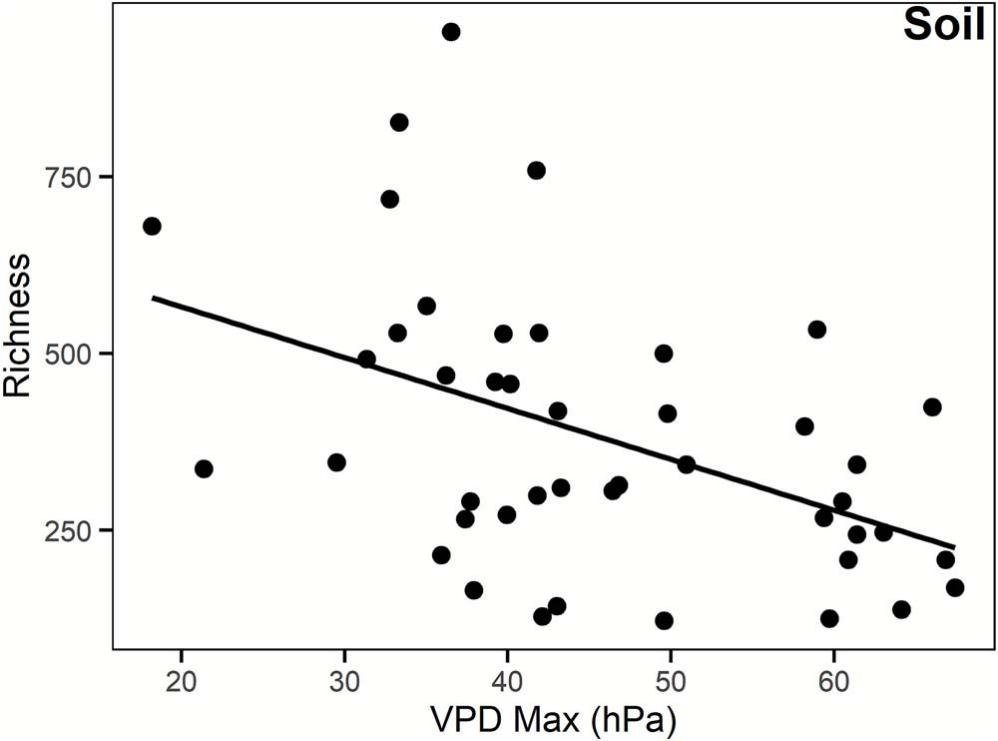
Relationships between soil fungal richness versus maximum VPD (*P* = .011). Symbols are sites. Lines denote the linear regression least squares best fit.

Fungal richness varied with geography across the Southwestern USA (Fig. 1). In soil, richness increased in areas with lower maximum VPD (*P* = .011, Table 1, Fig. 2). In air, richness was not significantly related to mean annual precipitation, mean annual temperature, maximum VPD, EVI, and land cover category. No other environmental variables were significant for airborne fungi.

**Figure 3:**
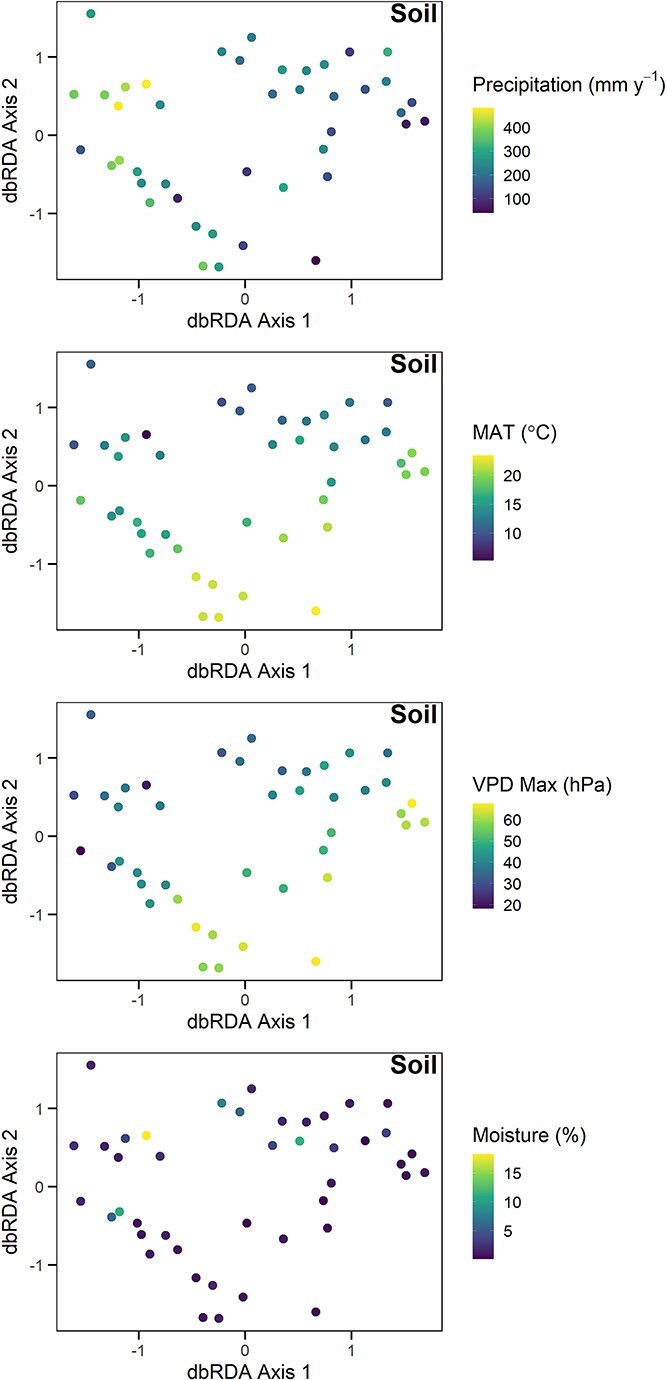
Multidimensional space (MDS) graphs of fungal community composition in soil. Symbols are sites. The proportion of variance explained by Axis 1 is 0.099 and by Axis 2 is 0.055. Symbol fill represents mean annual precipitation (*P* < .001), mean annual temperature (MAT, *P* = .006), maximum vapor pressure deficit (VPD max, *P* = .029), or soil moisture (*P* = .037). Sites that are closer to one another on the graph have a more similar community composition.

**Figure 4:**
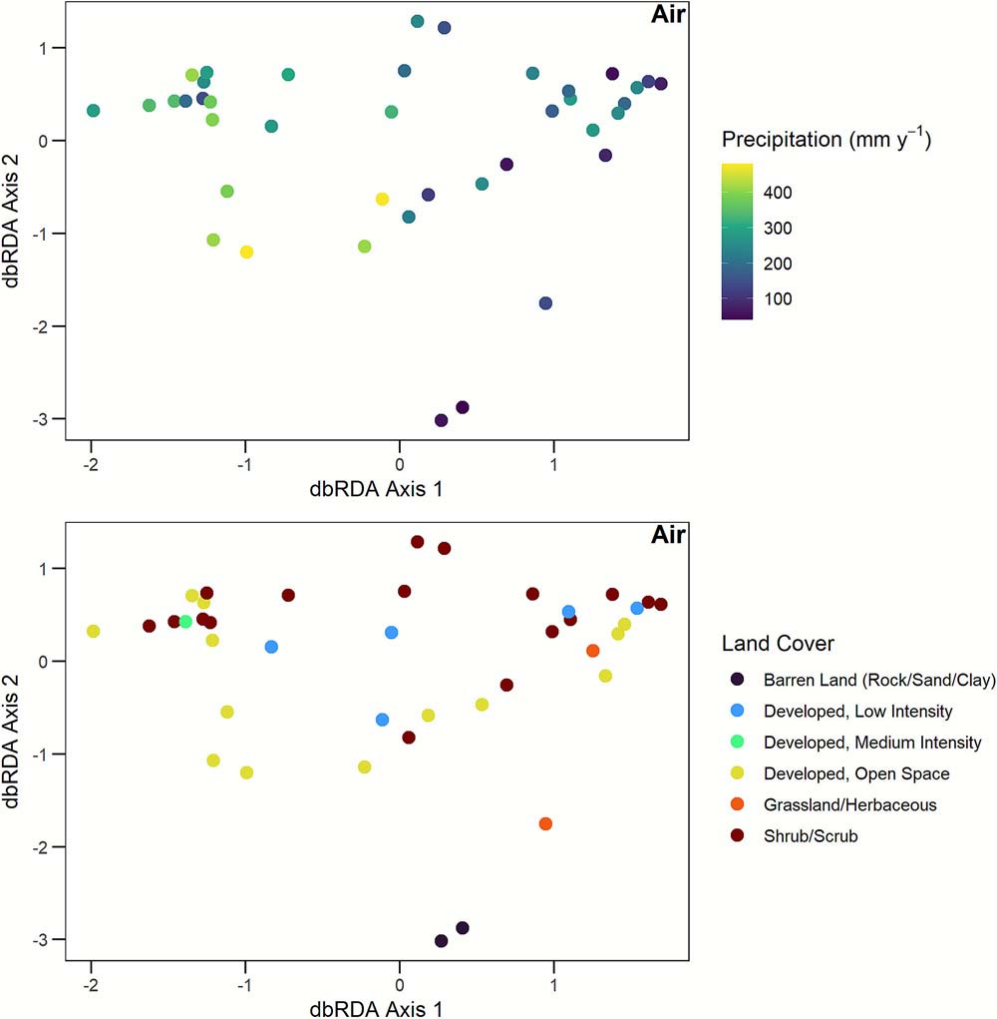
MDS graph of fungal community composition in air. Symbols are sites. Symbol fill represents precipitation (*P* = .008) and land cover category (*P* = .029). The proportion of variance explained by Axis 1 is 0.141 and by Axis 2 is 0.063. Sites that are closer to one another on the graph have more similar community composition.

Fungal community composition, based on Bray Curtis dissimilarities, also varied significantly with climate and vegetation (Table 1). Specifically, soil community composition was most significantly related to mean annual precipitation (Fig. 3, *P* < .001), followed by mean annual temperature (*P* = .006), maximum VPD (*P* = .029), and moisture (*P* = .037). In comparison, air community composition was significantly related to precipitation (Fig. 4, *P* = .008) and land cover category (*P* = .029). Altogether, Hypothesis 1 was supported for richness and community composition in soil and air, with the sole exception of air richness not varying significantly with climate or vegetation.

We investigated the fungal taxa that contributed most to regional community composition shifts as a function of precipitation. In soil, two taxa in the Agaricales (Agaricaceae), OTU129 and OTU55, and were the strongest contributors to shifts in response to precipitation (Table S2, Fig. S2). They increased in abundance with mean annual precipitation. In contrast, a species in the Pleosporaceae, OTU17409, decreased. In the air, two genotypes of *Alternaria alternata* (Pleosporaceae), OTU349 and SH429908.07FU_KF465761_refs, were the top contributors, and their abundance was positively related to precipitation (Table S2, Fig. S3). Conversely, a species in the Pleosporaceae, OTU513, decreased with precipitation.

For airborne communities, since land cover category was significantly related to community composition, we identified species that contributed most strongly to this pattern. The top three strongest contributors were OTU 9605 (*Cryptococcus paraflavus*), which was associated with grassland and herbaceous vegetation; OTU 284 (Nectriaceae species), associated with barren land; and OTU 61 (Ascomycota species), associated with low intensity development (Table S3, Fig. S4).

### Hypothesis 2: Community similarity and geographic distance

**Figure 5:**
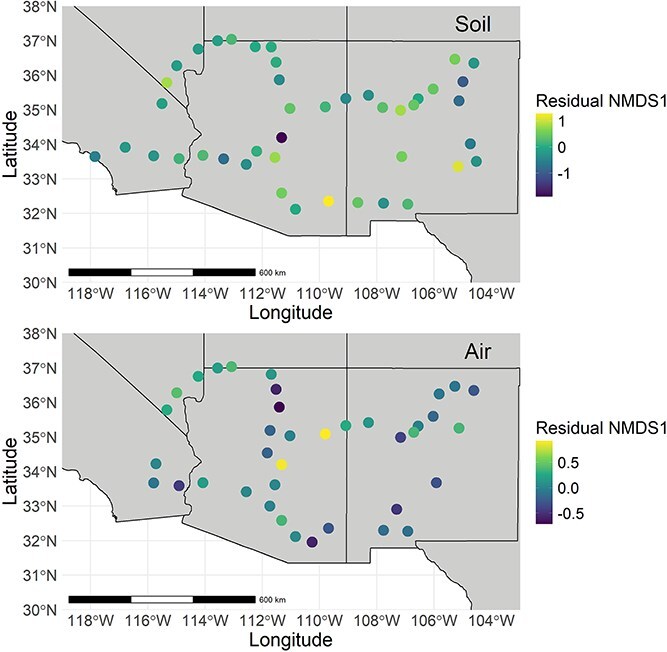
Residual NMDS1 maps of fungal community composition. Sites with similar residual NMDS1 values have similar fungal community compositions. Residual NMDS1 was normalized for climate and vegetation variables. Each symbol represents one site.

We used dbRDA to visualize shifts in fungal community composition across the Southwestern USA (Fig. 5). This analysis represented fungal community composition as NMDS axes. This approach normalized for mean annual precipitation, mean annual temperature, maximum VPD, EVI, and land cover category by calculating the residual NMDS values. In the residual NMDS1 maps, there was heterogeneity in community composition even between neighboring sites.

**Figure 6:**
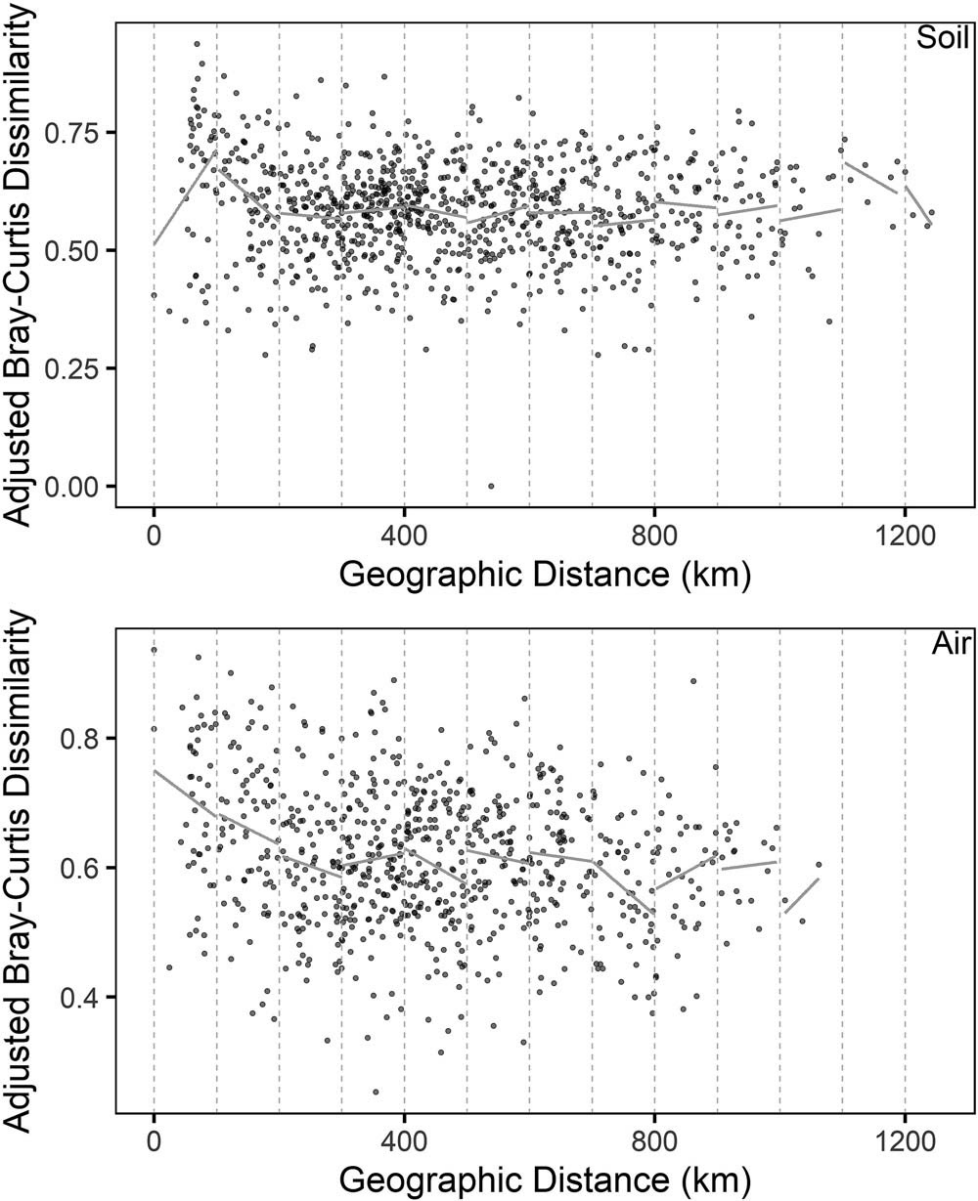
Mantel correlations between geographic distance versus fungal community dissimilarity for soil and airborne fungal communities. We normalized for mean annual precipitation when calculating community dissimilarity. Correlations were not significant for soil (Mantel *P* = .925) or air (*P* = .863). Symbols are pairs of sites. Reference lines indicate 100 km intervals. Grey solid lines are best fit relationship for site pairs within each 100 km interval, to facilitate comparisons with the Mantel correlogram (Fig. 7). Because we excluded samples with low numbers of DNA reads, air samples with distances >1100 km were not represented.

We used Mantel r tests to examine correlation between community dissimilarity and geographic distance between sites, while taking mean annual precipitation, mean annual temperature, and maximum VPD into account. When we used this approach to normalize for climate and vegetation, we found little evidence of spatial autocorrelation in air- or soilborne fungal communities (Fig. 6). Broadly, there were no significant correlations in soil (Mantel r = −0.105, *P* = .925) or air (Mantel r = 0.084, *P* = .863) across all sites. Neighboring sites did not tend to harbor more similar fungal communities than did distant sites.

**Figure 7:**
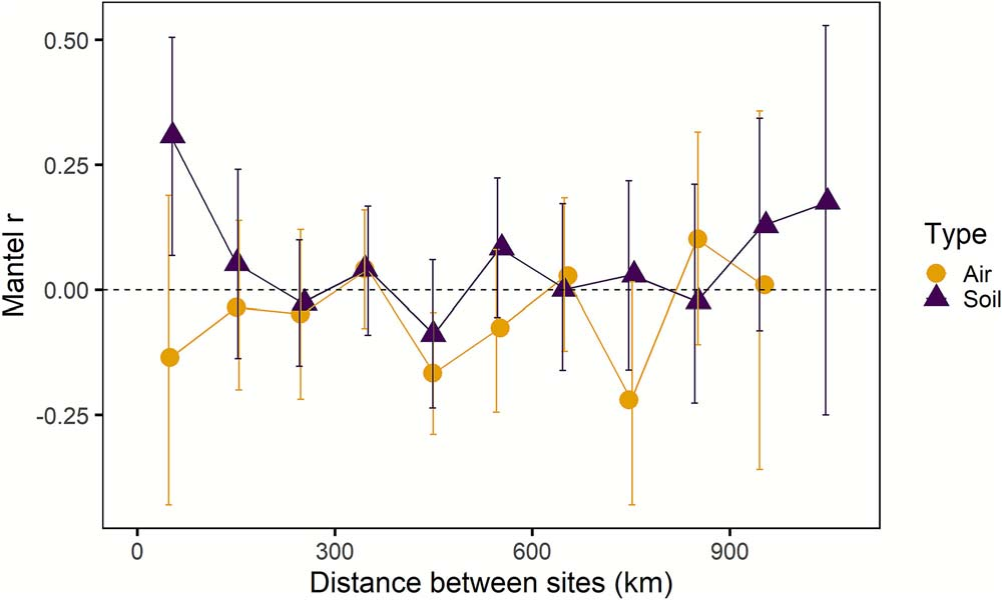
Mantel correlograms between fungal community dissimilarity and geographic distance in the soil and air. Each symbol represents the Mantel r correlation coefficient between pairs of sites that fall within the distance range. In sites that were between 0 and 100 km apart, soilborne fungal community dissimilarity was positively related to geographic distance. Each symbol represents 34 to 344 site pairs. Error bars are 95% confidence intervals. Symbols with error bars that do not overlap are significantly different from one another. Symbols are offset slightly to facilitate error bar visibility. Because we excluded samples with low numbers of DNA reads, air samples with distances >1100 km were not represented.

We examined in more detail the spatial scale of relationships between community dissimilarity and geographic distance to determine whether spatial autocorrelations were apparent at any scale. Soil communities were significantly autocorrelated only at the smallest scale: 0 to 100 km (Fig. 7). In addition, air communities displayed no significant autocorrelation. Altogether, we detected some support for Hypothesis 2, but only in soils at relatively short distances.

### Hypothesis 3: Soil versus air communities and geographic distance

Soil and air communities did not differ in degree of spatial autocorrelation when considering all sites in the transect (*P* = 1.000). In addition, soilborne fungi did not display significantly higher Mantel r statistics than did airborne fungi, at any scale (Fig. 7). We rejected Hypothesis 3.

## Discussion

As we hypothesized, fungal community composition in the soil and air in the Southwestern USA were each related to the environment, especially water availability (e.g. mean annual precipitation and maximum VPD; Table 1, Figs 3 and 4). When community composition was normalized for precipitation, only soilborne fungi exhibited spatial autocorrelation, and only at relatively short distances (<100 km, Fig. 7). In other words, we did not find strong evidence for dispersal limitation at the regional scale, especially in the air [57]. Altogether, fungal taxa may be affected if climate change reduces water availability in this dry region, and wind dispersal may facilitate fungal movement in response.

Our findings that environmental conditions related to fungal community composition in soil and air across a regional landscape are not unusual. For soils, in a global survey of drylands, Maestre and colleagues [6] noted that fungal diversity declined as aridity increased. They surmised that declining water availability tended to reduce soil organic matter, which may have limited energy use by fungi. Precipitation or temperature variables were also found to be related to soil fungal community composition in boreal, temperate, tropical, and subtropical ecosystems [31, 32, 34–37]. Likewise, for airborne fungi, a recent study reported that mean annual temperature was the strongest predictor of community composition at the global scale, with higher diversity nearer the equator [87]. Moreover, climate appears related to airborne fungal community composition in Croatia [88], Turkey [89, 90], and the Intermountain Western USA [91]. Climate may be an important driver of fungal biogeography regardless of biome.

In addition to climate, land cover type was linked to airborne community composition (Fig. 4). Specifically, the community composition in barren land (rock, sand, or clay) tended to differ from that of developed land and shrublands. The developed land included low and medium intensity (i.e. mixture of constructed materials and vegetation) and open space (i.e. areas with mostly lawn grasses). It is possible that a portion of the airborne community included leaf endophytes where vegetation was present [92]. For instance, the yeast *Cryptococcus paraflavus* (Cryptococcaceae) was the strongest contributor to variation among land cover categories, and it was most abundant in grasslands and herbaceous vegetation (Table S3, Fig. S4). This species has been isolated as an endophyte growing on leaves of a perennial grass in Spain [93].

By sampling fungal communities in the soil and air simultaneously, we can compare distance decay relationships between the two environments. Multiple mechanisms can influence distance decay relationships, including dispersal limitation, spatially structured environmental conditions, and species identity [57, 94–96]. The distance-based mapping approach allowed us to adjust distance decay relationships for spatial structure in mean annual precipitation. After accounting for mean annual precipitation, greater dispersal limitation of fungi should be accompanied by stronger distance decay relationships [57]. Accordingly, we had hypothesized that distance decay would be weaker in air than in soil, if wind facilitates dispersal. In soil, vectors of fungal dispersal can include plants, animals, water, or genet expansion [10, 97, 98]. Contrary to expectations, we found no significant spatial autocorrelation for airborne fungi, nor did the strength of their spatial autocorrelation differ from that of soilborne fungi (Figs 6 and 7). In fact, we did not find a difference between soil and air even though sampling techniques unavoidably differed between the two sample types. Accordingly, there was little evidence of dispersal limitation in this region.

*Alternaria* species were top contributors to shifts in airborne community composition with precipitation (Table S2). This genus is cosmopolitan and can wind disperse [99–104]. In Spain, asexual spores of *Alternaria* were frequently present in air samples, and were most abundant immediately after rainfall [99]. Airborne *A. alternata* increased significantly with precipitation (Fig. S3). This species functions as a pathogen, endophyte, and saprotroph [105–108]. Soft white spring wheat harbors a higher incidence of black point, a disease caused by *A. alternata*, where precipitation and irrigation are greater [109]. Likewise, rainfall is positively related to the incidence of Alternaria brown spot (caused by *A. alternata*) on citrus [110]. In contrast, endophytic *Alternaria* species colonizing the plant *Malva sylvestris* tended to increase with drought and may help confer drought tolerance to their host [111]. Our findings are likely context dependent.

For soilborne fungi, the top two contributors to shifts in community composition with precipitation belonged to the order Agaricales (Table S2, OTU 129, OTU 55). Both increased in relative abundance as precipitation increased (Fig. S2). About 40 000 species of Agaricales have been described [112, 113]. In addition, they are globally distributed [114–116]. Agaricales species can act as saprotrophs, mycorrhizal fungi, lichens, and pathogens [113, 117, 118]. Most produce gilled mushrooms (i.e. basidiocarps) [112], especially following precipitation [119].

In this study, we focused on spatial instead of temporal variation in fungal communities. We took this approach because regional-scale studies of airborne fungi are rare [56], and because it allowed us to estimate the scale of dispersal limitation [57]. Given time and financial constraints, this focus on extensive spatial sampling necessarily restricted our ability to take multiple samples over time. We emphasize that these samples represent a snapshot in time. Indeed, seasonal variation in airborne fungal communities is marked, especially between growing and non-growing seasons [87]. Had we sampled during cooler and wetter months, when plants can be more active in this dry region, perhaps airborne biogeography could have been more pronounced. Accordingly, we limit our interpretations to warmer and drier conditions in this region.

In conclusion, we conducted a regional-scale survey of soil and airborne fungi to ask how readily fungi might disperse in response to climate change. Precipitation seemed to be an important factor structuring fungal biogeography, given its relationship to fungal community composition in both soil and air. Since climate change is altering precipitation regimes in this region [14, 17], some fungal taxa may become vulnerable. Wind dispersal may allow a portion of the fungal community to follow their ecological niche within this region. Altogether, dispersal might be one way for fungi to respond to climate change in this dry region. Yet, it remains to be seen whether dispersing fungi can successfully establish at their destination.

## Supplementary Material

ycaf249_Supplemental_Files

## Data Availability

The datasets generated during and/or analyzed during the current study are available in the Figshare repository at 10.6084/m9.figshare.28611956 (R workflow) and https://doi.org/10.6084/m9.figshare.28611989.v1 (representative DNA sequences and taxonomy).
