# Supplementary material for: The biogeography of soil and airborne fungi in the Southwestern USA in relation to climate and vegetation
Source: ISME Commun. 2026 Jan 9;6(1):ycaf249. doi: 10.1093/ismeco/ycaf249 (PMC12855156; doi:10.1093/ismeco/ycaf249)
Supplement: ycaf249_Supplemental_Files [file ycaf249_supplemental_files.zip › Cat_etal_ms_ISMEComm_SupplMaterials_v3_ycaf249.docx]

**Table S2.** Taxa with the greatest contribution to changes in community composition with precipitation.

| Taxon ID | Name | IndVal statistic | P |
| --- | --- | --- | --- |
| Soil |  |  |  |
| OTU129 | Agaricales | 0.823 | 0.006 |
| OTU55 | Agaricales | 0.796 | 0.002 |
| OTU17409 | Pleosporaceae species | 0.789 | 0.010 |
|  |  |  |  |
| Air |  |  |  |
| OTU349 | *Alternaria alternata* | 0.809 | 0.004 |
| SH429908.07FU_KF465761_refs | *Alternaria alternata* | 0.799 | 0.002 |
| OTU513 | Pleosporaceae species | 0.693 | 0.018 |

**Table S3.** Taxa with the greatest contribution to changes in community composition with land cover category in air samples.

| Taxon ID | Name | IndVal statistic | P |
| --- | --- | --- | --- |
| OTU 9605 | *Cryptococcus paraflavus* | 0.956 | 0.002 |
| OTU 284 | Nectriaceae species | 0.698 | 0.044 |
| OTU 61 | Ascomycota species | 0.697 | 0.032 |


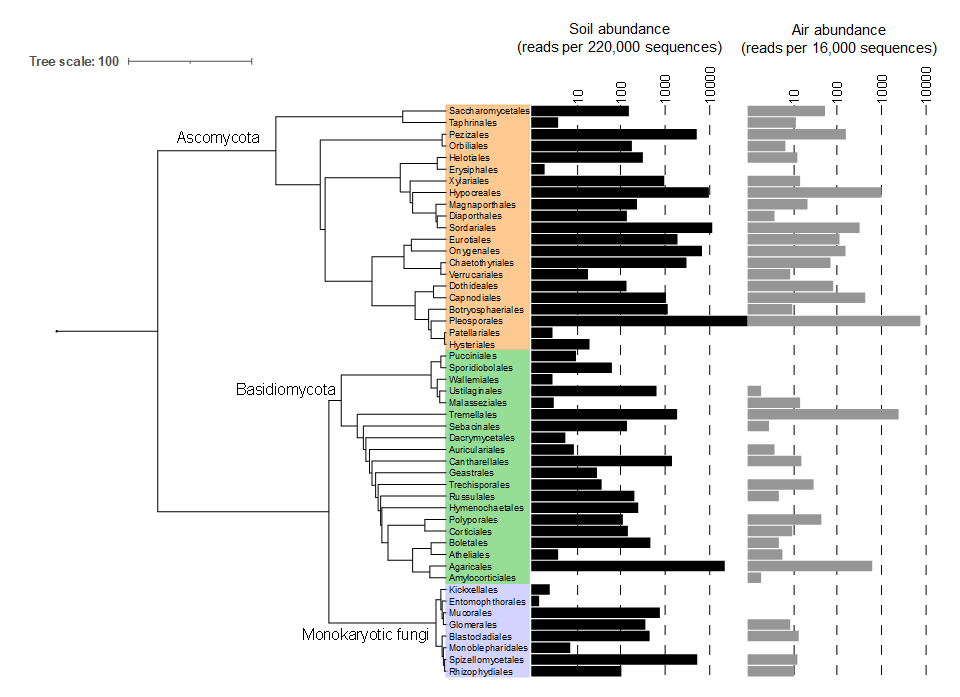


Figure S1. Phylogeny of fungal orders represented in the study. Relative abundance of each order is represented by black (soil) or grey (air) bars. Note log axis.


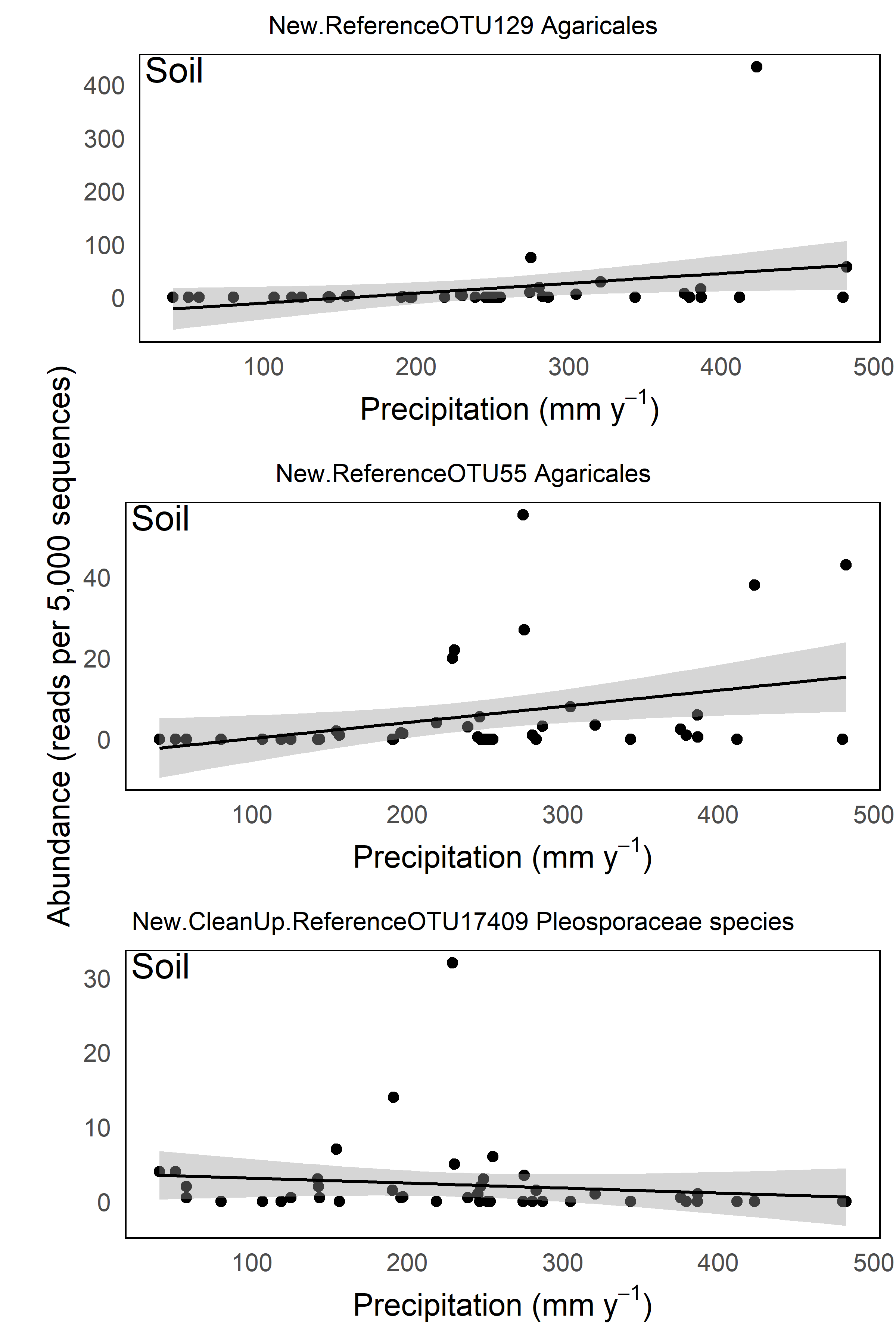


Figure S2. Relationships between relative abundance and precipitation of the three most significant indicator taxa in soil samples in the Southwestern U.S. Symbols are sites. Lines are best fit.


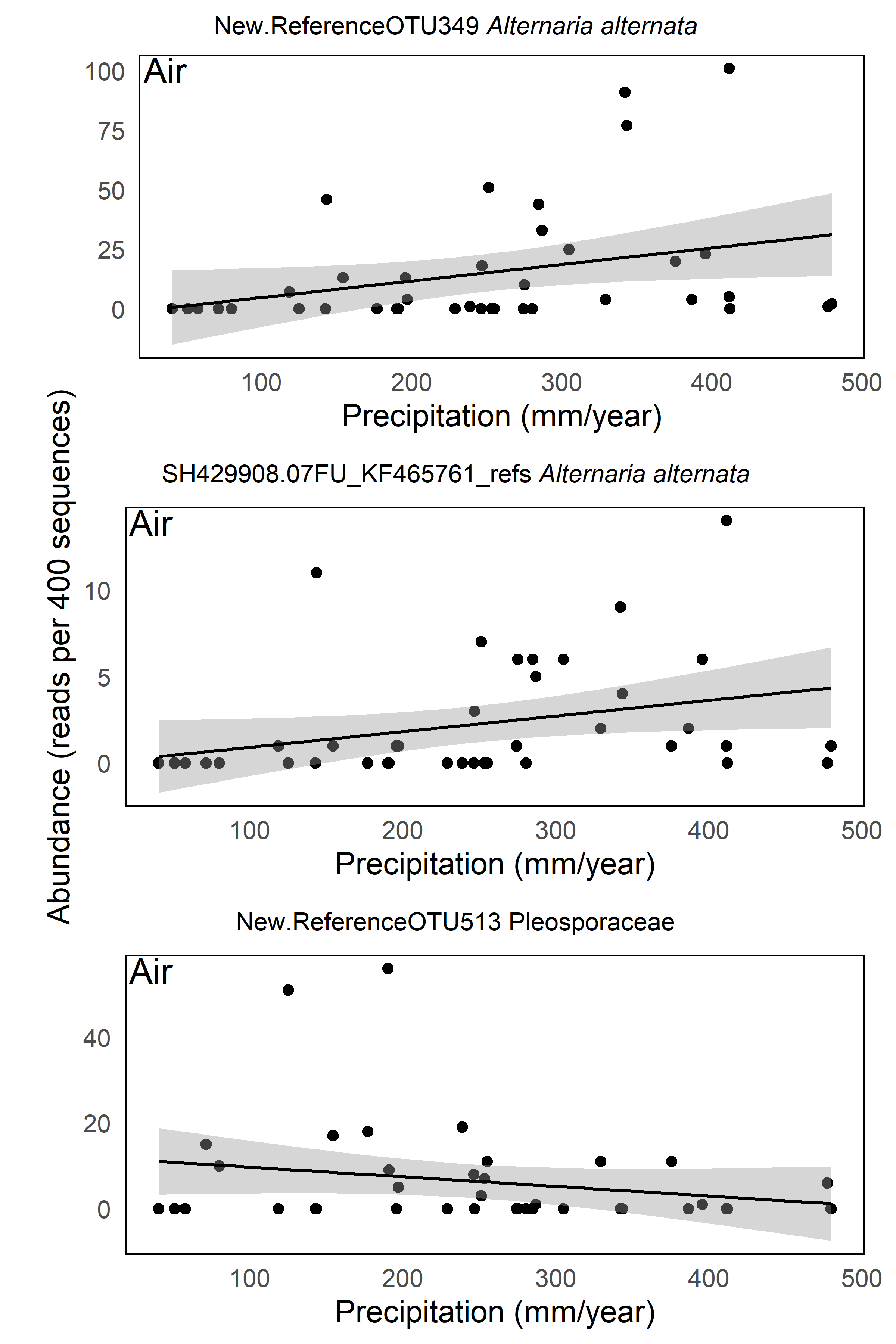


Figure S3. Relationships between relative abundance and precipitation of the three most significant indicator taxa in air samples in the Southwestern U.S. Symbols are sites. Lines are best fit.


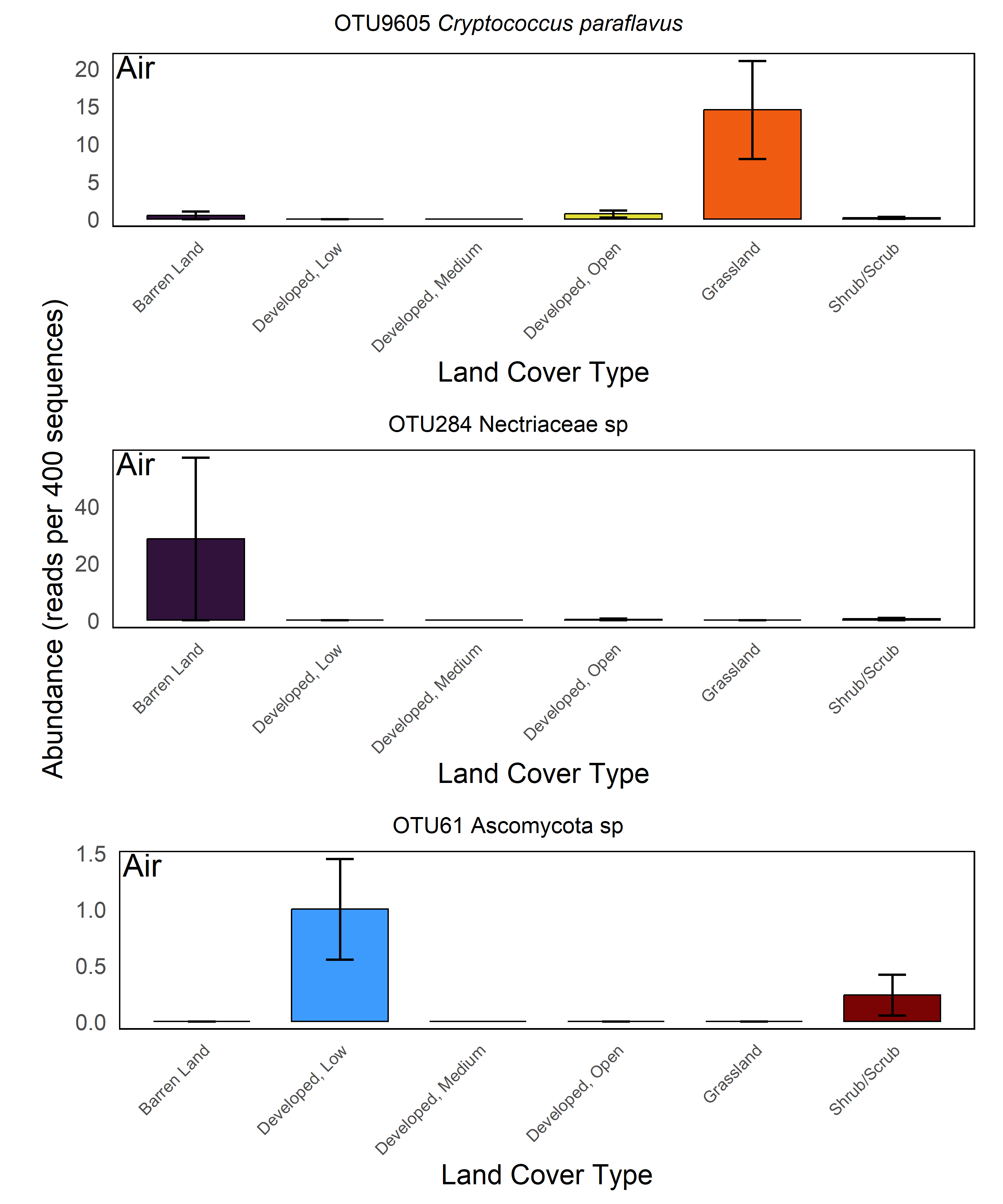


Figure S4. Relationships between relative abundance and land cover category of the three most significant indicator taxa in air samples in the Southwestern U.S. Symbols are sites. Bars are means ±1 SE.
